# Diagnosis and treatment of leg-length discrepancy in scoliosis

**DOI:** 10.1186/1748-7161-8-S2-O41

**Published:** 2013-09-18

**Authors:** Franz Landauer

**Affiliations:** 1 Univ. Clinic of Orthopedics (PMU), Salzburg, Austria

## Background

Balancing the sacrum is a prerequisite to the treatment of scoliosis. 

## Purpose

The purpose of this study was to diagnose the causes of leg-length discrepancy and review their surgical treatment options. 

## Methods

The x-rays (upright ap-view) of 250 patients diagnosed with “idiopathic scoliosis” were examined to determine a leg length difference of >1.0 cm. First issue: How many patients show scoliosis as a compensation mechanism of leg-length discrepancy? How many needed epiphysiodesis in differences >1.5cm. Second issue: What causes of leg-length discrepancy are found and what are the therapeutic consequences? Patients with neurological findings were excluded. 

## Results

*First issue: *In 5 of the 250 examined patients, scoliosis was only a compensation mechanism of the leg-length discrepancy. Leg-length compensation (insole) was sufficient for spinal correction. In three cases with a leg-length difference >1.5 cm, a temporary epiphysiodesis was indexed. *Second issue:* Two patients with pathological findings in the lower extremity showed hypoplasia of the fibula (type IA Achtermann and Kalamachi) and one patient showed a hypoplastic femur (type IX by Pappas). One patient had a femoral fracture in early childhood, one patient suffered from an injury of the femoral growth plate and one patient showed a fibrous dysplasia. In two cases, involving the hip, a chronic slipped capital femoral epiphysis was found requiring surgical intervention. Additional hormonal causes, such as Hashimoto thyroiditis and delayed puberty, were conspicuous. In one patient, a femoral head deformity after SCFE was diagnosed and corrected. One undiagnosed Perthes disease was found. Four patients with first diagnosed neurological findings are excluded. In 15 of 246 patients, leg-length-discrepancy >1cm was diagnosed. 

## Conclusions and discussion

Of the 246 patients, the identified 15 cases are a challenge to treating “scoliosis“ because the cause, or one of the causes, of scoliosis was found to be independent of the spine and had to be dealt with as such. With correct treatment of these cases, a partial correction of the ”scoliosis“ happens automatically. This study provides an initial overview of leg-length discrepancy and recommends: “Take the lower extremities into account when diagnosing and treating scoliosis.” .
